# Clinical Implications of Polyploid Giant Cancer Cells in Solid Tumors: Biology, Diagnosis, and Therapeutic Considerations

**DOI:** 10.3390/cancers18111818

**Published:** 2026-06-01

**Authors:** Hiroshi Imaoka, Masafumi Ikeda, Masashi Wakabayashi, Kumiko Umemoto, Tomoyuki Satake, Yu Sunakawa, Hideki Ueno, Kazuo Hara, Fumio Nagashima, Shigeki Kataoka, Terumasa Hisano, Yuko Suzuki, Akinori Asagi, Kazuhiko Shioji, Kotoe Oshima, Kunihiro Tsuji, Kazuyoshi Ohkawa, Ikuya Miki, Yasuyuki Kawamoto, Taro Yamashita, Makoto Ueno, Yujiro Kawakami, Hiroaki Nagano, Hiroyuki Okuyama, Atsushi Naganuma, Rei Suzuki, Junji Furuse

**Affiliations:** 1 Department of Hepatobiliary and Pancreatic Oncology, National Cancer Center Hospital East, Kashiwa 277-8577, Japan; 2 Clinical Research Support Office, National Cancer Center Hospital East, Kashiwa 277-8577, Japan; 3 Department of Clinical Oncology, St. Marianna University School of Medicine, Kawasaki 216-8511, Japan; 4 Department of Hepatobiliary and Pancreatic Oncology, National Cancer Center Hospital, Tokyo 104-0045, Japan; 5 Department of Gastroenterology, Aichi Cancer Center Hospital, Nagoya 464-8681, Japan; 6 Department of Medical Oncology, Kyorin University Faculty of Medicine, Tokyo 181-8611, Japan; 7 Department of Medical Oncology, Graduate School of Medicine, Kyoto University, Kyoto 606-8501, Japan; 8 Department of Hepato-Biliary-Pancreatology, National Hospital Organization Kyushu Cancer Center, Fukuoka 811-1347, Japan; 9 Department of Gastroenterology, Saitama Cancer Center, Saitama 362-0806, Japan; 10 Department of Gastrointestinal Medical Oncology, National Hospital Organization Shikoku Cancer Center, Matsuyama 791-0245, Japan; 11 Department of Internal Medicine, Niigata Cancer Center Hospital, Niigata 951-8566, Japan; 12 Division of Gastrointestinal Oncology, Shizuoka Cancer Center, Shizuoka 411-8777, Japan; 13 Department of Gastroenterology, Ishikawa Prefectural Central Hospital, Kanazawa 920-8530, Japan; 14 Department of Hepatobiliary and Pancreatic Oncology, Osaka International Cancer Institute, Osaka 541-8567, Japan; 15 Department of Gastroenterological Oncology, Hyogo Cancer Center, Akashi 673-8558, Japan; 16 Division of Cancer Center, Hokkaido University Hospital, Sapporo 060-8648, Japan; 17 Department of Gastroenterology, Kanazawa University Hospital, Kanazawa 920-8641, Japan; 18 Department of Gastroenterology, Kanagawa Cancer Center, Yokohama 241-8515, Japan; 19 Department of Gastroenterology and Hepatology, Sapporo Medical University School of Medicine, Sapporo 060-8556, Japan; 20 Department of Gastroenterological, Breast and Endocrine Surgery, Yamaguchi University Graduate School of Medicine, Ube 755-8505, Japan; 21 Department of Medical Oncology, Kagawa University Hospital, Miki 761-0793, Japan; 22 Department of Gastroenterology, National Hospital Organization Takasaki General Medical Center, Takasaki 370-0829, Japan; 23 Department of Gastroenterology, School of Medicine, Fukushima Medical University, Fukushima 960-1295, Japan

**Keywords:** polyploid giant cancer cell, polyaneuploid cancer cell, pleomorphic cancer cell, osteoclast-like giant cell, multinucleated giant cancer cell, undifferentiated carcinoma, cancer-associated macrophage-like cell, polyploidization, dormancy

## Abstract

Polyploid giant cancer cells (PGCCs) are characterized by abnormal enlargement and considerable polyploidy. Though the presence of giant cancer cells has been documented for decades, they remain not fully understood, especially in clinical practice, due to diagnostic challenges, and confusion regarding synonyms for PGCCs still exists. Thus, understanding PGCCs may be a key clue to overcoming them. This review offers a comprehensive overview of PGCCs, integrating insights from basic research and clinical studies to enhance understanding of their complex biology and clinical implications. In basic research, PGCCs are known to emerge under various stressors, including chemotherapy exposure, radiation, viral infection, and hypoxic environments. These cells play crucial roles in tumor progression through multiple mechanisms: enhancing genetic diversity, and facilitating metastatic spread via asymmetrical cell division and genomic instability. In clinical studies, PGCC-containing tumors have been shown to exhibit marked treatment resistance and are associated with a poor prognosis across multiple solid tumor types, including prostate, lung, and pancreatic cancers. Despite these therapeutic challenges, taxane-based chemotherapy has shown promising results in PGCC-containing tumors, such as pleomorphic carcinoma and undifferentiated carcinoma. Furthermore, emerging targeted therapies directed at specific pathways in PGCCs, particularly those involving TP53, represent potential strategies to improve clinical outcomes of patients with PGCC-containing tumors.

## Simple Summary

Polyploid giant cancer cells (PGCCs) are characterized by abnormal enlargement and considerable polyploidy. Although these cells have been documented in tumors for decades, they remain poorly understood, especially in clinical practice, due to diagnostic challenges and confusion regarding terminology for PGCCs. Emerging evidence suggests that PGCCs play crucial roles in tumor progression, facilitating metastatic spread via asymmetrical cell division and genomic instability. Their presence in tumor tissue has been associated with marked treatment resistance and a poor prognosis across multiple solid tumor types, suggesting their potential utility as a prognostic indicator. Although no established treatments exist, emerging targeted therapies targeting specific pathways in PGCCs represent potential strategies to improve clinical outcomes for patients with PGCC-containing tumors. This review integrates insights from basic research and clinical studies to enhance understanding of the complex biology and clinical implications of PGCCs.

## 1. Introduction

Polyploid giant cancer cells (PGCCs) are a type of cancer cell observed in various cancers, including prostate, lung, and pancreatic cancers. These cells arise through various mechanisms, including cytokinesis failure, cell fusion, and endoreduplication, leading to genomic instability and chromosomal aberrations [1]. Morphologically, the nuclei of PGCCs are usually irregular and large; Zhang defined PGCC nuclei as being ≥ three times greater than regular-sized diploid tumor cell nuclei [2].

Across all types of cancer, PGCCs are thought to be associated with more aggressive behavior and a worse prognosis. One reason is the cancer stem cell-like characteristics of PGCCs. Normal cells proliferate by symmetrical division, but PGCCs generate progeny cells through asymmetrical division other than normal mitosis. PGCCs can express cancer stem cell-related markers (CD44 and CD133) [3] and epithelial–mesenchymal transition (EMT)-related proteins to promote invasion and migration [4]. In addition, PGCCs can be related to chemoresistance and tumor recurrence. These characteristics resemble cancer stem cells [5] and are thought to contribute to the malignant phenotype of PGCCs.

PGCCs have become an increasingly important focus in translational research. However, their clinical significance is not well recognized in clinical practice. Though PGCCs are not uncommon in tumors, they typically represent only a small fraction of the total cell population [6,7]. This characteristic makes clinical diagnosis challenging and hinders understanding of PGCC biology and their potential therapeutic applications. This comprehensive review of current knowledge about PGCCs first provides an overview of PGCC biology in basic research, followed by an examination of their clinical implications based on clinical studies.

## 2. Terminology and Definitions of PGCCs

### 2.1. Definition Framework

This review adopts a two-tiered approach to defining PGCCs. In the narrow sense, PGCCs are defined as cancer cells with nuclei ≥ three times larger than regular-sized diploid tumor cell nuclei (Zhang’s definition) and polyploid DNA content confirmed by appropriate analytical methods. Following established practice in the literature [8], hyperdiploid and polyploidy are treated as overlapping concepts, since these terms have been used with some interchangeability in many studies, particularly when referring to cancer cell subpopulations with elevated genomic content relative to tumor bulk.

To broaden the scope of this review, the definition was expanded to include related entities with clinical significance. Since most aneuploid tumors exhibit chromosomal gains [9], aneuploidy is considered near-polyploid for practical purposes. Therefore, the present analysis encompasses not only classical polyploid cells, but also polyaneuploid cancer cells with complex chromosomal alterations and pleomorphic giant cancer cells with variable ploidy status. This broad approach reflects clinical reality, in which pathologists encounter a spectrum of morphologically enlarged cancer cells sharing common characteristics including treatment resistance, aggressive behavior, and poor prognosis.

Whereas this review focuses primarily on PGCCs in solid tumors, where their distinct morphological and functional characteristics have been more extensively investigated, polyploidy is not limited to solid tumors. Tetraploid/near-tetraploid acute myeloid leukemia and polyploid diffuse large B-cell lymphoma have also been associated with a poor prognosis [10,11]. Although the morphological features and underlying biological mechanisms may differ from those in solid tumors, these findings support the notion that genomic polyploidization represents a common pathway contributing to tumor progression and therapeutic resistance across diverse cancer types.

### 2.2. Terminology Confusion in the Literature

**Figure 1:**
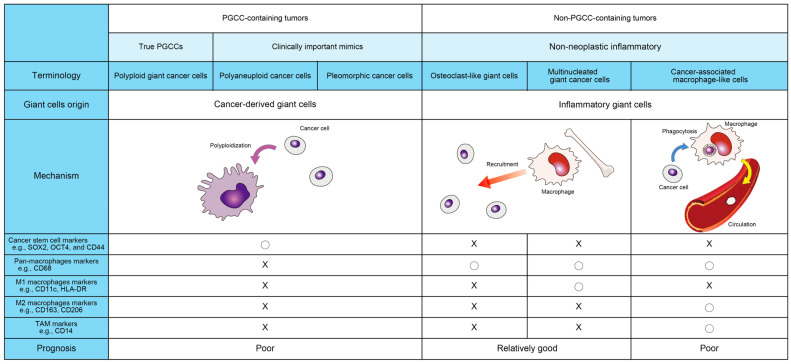
Key differences between cancer-derived giant cells (e.g., PGCCs and their mimics) and inflammatory giant cells. Five giant cell subtypes are contrasted by cellular origin, proposed mechanism, immunohistochemical profile, and prognosis. Diagnostically, the combination of cancer stem cell marker positivity (e.g., SOX2, OCT4, and CD44) with pan-macrophage marker (CD68) negativity is the strongest evidence supporting a true PGCC identity, whereas CD68 positivity with the absence of cancer stem cell markers indicates an inflammatory giant cell origin. Suggestive but not solely diagnostic features include M1 (CD11c, HLA-DR), M2 (CD163, CD206), and TAM (CD14) marker profiles, which show considerable overlap among subtypes, as well as clinical prognosis. ○, positive; ×, negative. TAM, tumor-associated macrophage.

The terminology for PGCCs has varied, and polyaneuploid cancer cells, pleomorphic cancer cells, osteoclast-like giant cells (OGCs), cancer-associated macrophage-like cells, and multinucleated giant cancer cells are often regarded as synonymous [12]. However, the diagnoses for these terms were based solely on morphological findings, so confusion exists (Figure 1).

Polyaneuploid cancer cells and pleomorphic cancer cells are considered cancer-derived giant cells synonymous with PGCCs. Polyaneuploid cancer cells are known to have undergone whole-genome duplication, resulting in at least twice the complement of the original aneuploid genomic content. These unusually large aneuploid cancer cells have been well documented in the cancer literature since 1858, when Virchow first described them [13]. These cells exhibit characteristics similar to those of PGCCs in terms of biological behavior and clinical implications [12], and they are considered synonymous with PGCCs. This entity has been well documented in the prostate.

Pleomorphic carcinoma of the lung is a rare, poorly differentiated non–small cell lung cancer (NSCLC) that contains at least 10% spindle and/or giant cells and is currently categorized as a subtype of sarcomatoid carcinomas of the lung by the WHO classification of thoracic tumors [14]. These spindle and giant tumor cells are now considered part of the same clonal neoplasm.

**Figure 2:**
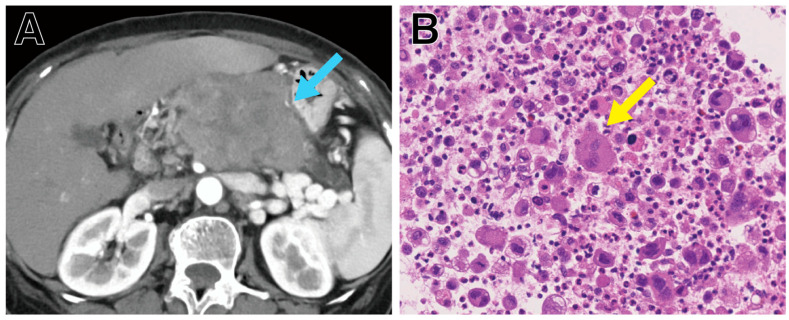
Imaging and histological findings of PGCC-containing tumors of the pancreas (undifferentiated carcinoma defined by the WHO classification). (**A**) Contrast-enhanced computed tomography demonstrates expansive tumor growth pattern of the PGCC-containing tumor (blue arrow), contrasting with the typical infiltrative pattern of conventional pancreatic cancer. (**B**) Hematoxylin and eosin staining reveals PGCCs (yellow arrows) with enlarged nuclei and abundant cytoplasm, larger (≥3× nuclear diameter) than surrounding conventional cancer cells, displaying characteristic polyploid morphology.

In the pancreas, pleomorphic carcinoma, also known as anaplastic carcinoma, is a rare subtype of undifferentiated carcinoma. The tumors consist of pleomorphic mononuclear cells admixed with bizarre-appearing giant cells with eosinophilic cytoplasm and lack gland formation [15] (Figure 2). These PGCC-containing tumors are reported to be markedly treatment-resistant and associated with a poor prognosis (see Section 6. Clinical Implication of PGCCs for further details).

In contrast, OGCs, multinucleated giant cancer cells, and cancer-associated macrophage-like cells consist of non-neoplastic cells, which are considered inflammation-derived giant cells. These giant cells are all thought to be derived from macrophages and have been induced in response to tumors. The cells are morphologically similar but different entities from PGCCs.

**Figure 3:**
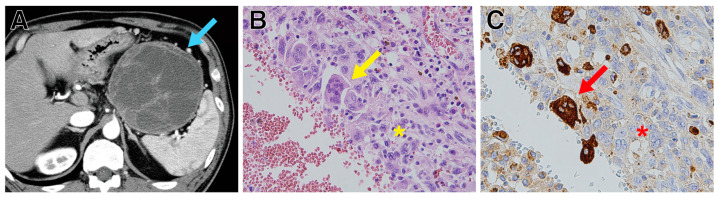
Imaging and histopathologic findings of osteoclast-like giant cells of the pancreas (undifferentiated carcinoma with osteoclast-like giant cells defined by the WHO classification). (**A**) Contrast-enhanced computed tomography demonstrates a large tumor with round contour and marked cystic degeneration in the pancreatic tail (blue arrow). (**B**) Hematoxylin and eosin staining shows a mixture of pleomorphic mononuclear tumor cells (yellow asterisk) and non-neoplastic osteoclastic multinucleated giant cells (yellow arrow). (**C**) Pleomorphic mononuclear tumor cells are negative for CD68 (red asterisk), while multinucleated osteoclast-like giant cells are positive (red arrow). [(**B**,**C**) represent consecutive sections from the same tissue area].

OGCs have been documented in various organs, particularly the pancreas, and they are classified as undifferentiated carcinoma with OGCs. Pathologically, they contain neoplastic pleomorphic cells (high Ki-67 index, positive for epithelial markers) and multi-nucleated OGCs (few mitoses, positive for macrophagic marker CD68, negative for epithelial markers) (Figure 3). These findings suggest that OGCs are reactive cells from macrophages rather than neoplastic cells. Studies by Strobel et al. and Muraki et al. indicate that patients with undifferentiated carcinoma with OGCs have significantly longer overall survival (OS) than those with undifferentiated carcinoma or conventional pancreatic cancer [16,17].

Cancer-associated macrophage-like cells (CAMLs) are generally larger than circulating tumor cells, ranging from 25 to 300 µm. They have atypical or multiple nuclei and contain phagocytosed tumor protein epitopes in the cytoplasmic vesicles. Since CAMLs express the macrophage protein CD14 on their cell surfaces, they are believed to represent tumor-associated macrophages that have phagocytosed tumor cell material locally and have disseminated [18]. The prognostic significance of circulating CAMLs has also been reported in various types of cancers [19]. In a study by Adams et al. [18], circulating CAMLs were detected in 93% of solid tumor patients, but not in healthy volunteers; furthermore, tumor cell diameter and polyploidy increased with tumor progression. Large CAMLs (>50 µm, particularly >110 µm) were significantly associated with poor progression-free survival (PFS) and overall survival (OS). The authors suggested that tumor microenvironmental stimuli induce polyploidization of myeloid-derived hematopoietic stem cells and monocytic cells into PGCCs, a fraction of which disseminate into the circulation as CAMLs.

Multinucleated giant cells typically occur in granulomatous diseases, including infections, vasculitis, immunological disorders, and cancer. They are formed by the fusion of circulating monocytes, progenitors of macrophages, infiltrating into local tissues. These cells display surface markers (CD11c+, HLA-DR+, CD163-, CD206-) similar to M1 macrophages, indicating that these cells are anti-tumor M1 macrophages [20,21].

## 3. Polyploidization and Dormancy

**Figure 4:**
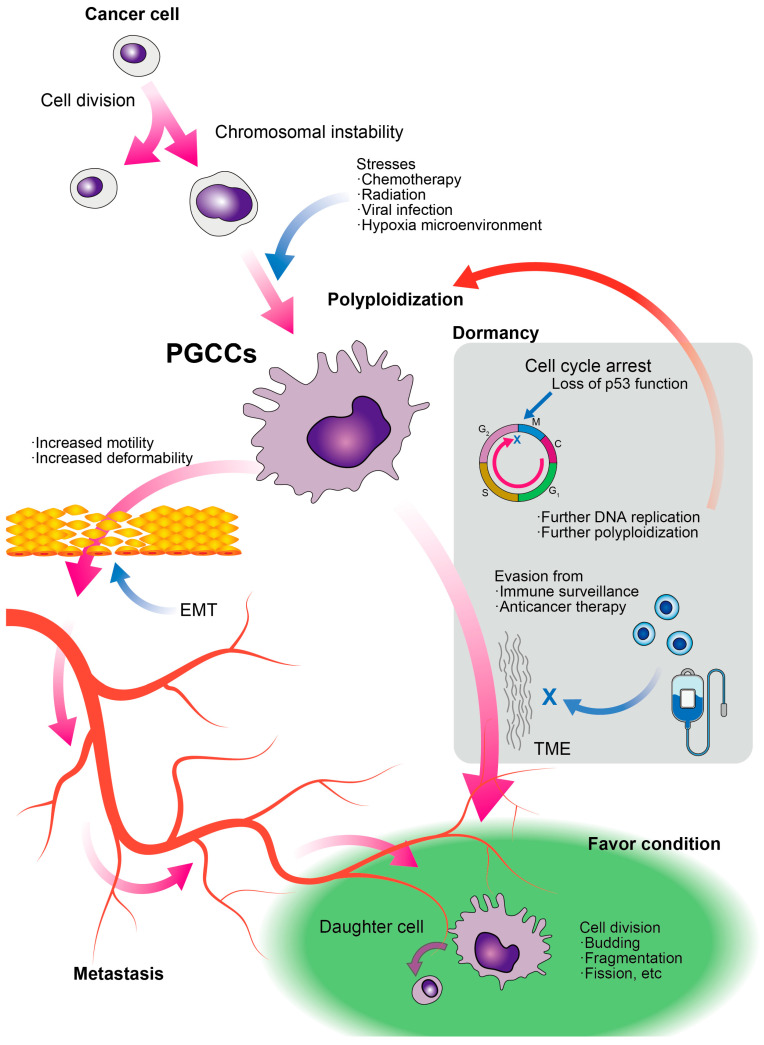
Comprehensive overview of the formation and functions of PGCCs. PGCCs, polyploid giant cancer cells; EMT, epithelial–mesenchymal transition; TME, tumor microenvironment.

The multinucleated morphology of PGCCs is considered the outcome of polyploidization and dormancy. PGCCs undergo polyploidization and enter a dormant state in response to stressors such as chemotherapy, radiation, viral infection, and a hypoxic microenvironment [3,22,23]. Consequently, polyploidy and dormancy cause a poor prognosis for tumors by promoting tumor evolution through the accumulation of chromosomal instability, acquisition of therapeutic resistance, and metastasis (Figure 4).

### 3.1. Polyploidization

Polypoidization is the process by which a cell gains one or more extra sets of chromosomes, leading to polyploidy, which is a genetic condition in which cells have more than two complete sets of chromosomes. Normal cells have two sets of chromosomes (2n), with one set inherited from each parent (diploid). In contrast, polyploid cells have three or more complete sets of chromosomes (3n, 4n, etc.). These cells are common in plants but are uncommon in most humans except for specific human cell types: hepatocytes (4n) and megakaryocytes (up to 64n). In cancers, polyploidization is a relatively common phenomenon and plays a significant role in tumor evolution and progression.

In the nuclei of PGCCs, increased DNA content has been observed using various DNA analysis techniques [24]. Pan-cancer genomic analysis has shown that a high proportion of human solid tumors (28.2–37%) have experienced at least one round of polyploidization during their evolution [25,26]. Chromosome missegregation during cell division leads to uneven distribution of chromosomes between daughter cells. Such polyploidization promotes chromosomal instability, an abnormality in chromosomal number or structure, which in turn facilitates cancer evolution by generating genetic diversity [27]. Thus, polyploidy was considered to be mainly caused by an abnormality in cell division. However, recent studies suggest that polyploidization can occur early in cancer development after oncogenic driver events, such as TP53 dysfunction and cell-cycle arrest (see Section 3.2. Dormancy for further details) [25].

### 3.2. Dormancy

Dormancy is a temporary, reversible state in which cells stop dividing and enter quiescence while remaining viable. Simply put, cells go into a “sleep mode” in which they maintain minimal metabolic activity, but they can “wake up” and resume normal function when conditions become favorable. Dormant cancer cells share several identical characteristics and signaling pathways with cancer stem cells, including the ability to metastasize and evade immune surveillance [28,29]. To maintain dormancy, tumor cells suppress genes required for DNA synthesis or cell division and promote the expression of anti-apoptotic, anti-aging, and anti-differentiation genes [30,31]. Dormancy can allow cancer cells to undergo different cellular states (migration, quiescence) and evade anticancer therapies [32,33,34]. Dormant PGCCs remain in a non-proliferative state, evading therapeutic effects, and later reactivate and proliferate, leading to tumor recurrence [23].

PGCCs have two major characteristics similar to dormant cancer cells: cell-cycle arrest and high migratory ability. Cell-cycle arrest is caused by disruption of the cell-cycle checkpoint. In normal cells, this cell-cycle checkpoint monitors the process of the cell cycle and pauses or inhibits its progression if abnormalities are detected. This checkpoint mechanism is disrupted in PGCCs, and the cell cycle is arrested [35,36]. Essentially, cell-cycle arrest refers to the halting of the cell cycle and DNA replication in response to detected damage, allowing time for repair before mutations can occur. However, during cell-cycle arrest in PGCCs, these cells undergo further DNA replication and polyploidization in the nucleus, known as endoreplication. They are survival strategies dormant PGCCs adopt to adapt to harsh environmental conditions. Cell-cycle arrest in PGCCs consists of the following four steps [37]. First, multiple stresses can induce cell-cycle arrest in cancer cells, leading to the formation of PGCCs for survival after anti-mitotic treatment [38,39]. Second, PGCCs replicate chromosomes without mitosis, which allows tumor cells to grow in a polyploid state. In this state, PGCCs evade this checkpoint through loss of p53 function and continue proliferating even with abnormal DNA levels [38,39]. Third, PGCCs generate daughter cells distinct from conventional mitosis through processes via alterations in aurora kinases A and B, which regulate chromosome segregation: budding (where small cells emerge from the PGCC surface and eventually separate to form independent daughter cells), fragmentation, and fission (where PGCCs divide their nuclear content into smaller nuclei, facilitating cellular division) [38,40]. Furthermore, PGCCs activate autophagy pathways to maintain survival and proliferative capacity under stressful conditions. Finally, daughter cells acquire new genomes and resume mitosis [38,40].

Another major characteristic of PGCCs similar to dormant cancer cells is their high migratory ability. PGCCs exhibit increased motility and deformability mediated by icreased mesenchymal-related protein expression and show increased metastatic potential. It has been reported that PGCCs have thicker, longer actin fibers and abnormal overexpression of actin components compared with diploid cancer cells [41]. This mechanism allows PGCCs to be highly motile and metastatic. Furthermore, dormancy of tumor cells is an essential stage of tumor metastasis, since it allows dormant cells to survive during dissemination, remain dormant until favorable conditions appear, and adapt to circumstantial conditions. Dormant PGCCs can be activated by external signals such as growth factors and cytokines from the microenvironment [42]. These signals promote cell proliferation, causing dormant tumor cells to re-enter the cell cycle and initiate metastasis [43].

PGCCs exit dormancy and produce daughter cells via asymmetric division. However, this asymmetric division leads to the loss of genetic material in the mononuclear daughter cells, thereby increasing their genetic instability and heterogeneity [2]. Metastatic capabilities of PGCCs and their daughter cells, including proliferation, migration, and invasion, are enhanced through high expression of EMT-related proteins [44]. Vimentin is highly expressed in both PGCCs and their daughter cells, facilitating their migration and invasion abilities. This elevated vimentin expression enables these cells to metastasize to lymph nodes and distant organs while promoting migratory persistence [45].

### 3.3. Cell Sencescence

This dormancy–reactivation cycle shares mechanistic features with cellular senescence, an emerging concept in cancer biology in which cells undergo growth arrest while remaining metabolically active. Under normal conditions, senescence functions as a tumor-suppressive barrier through p53-mediated cell-cycle arrest and activation of anti-tumor immune responses via the senescence-associated secretory phenotype [46,47]. However, in the tumor microenvironment, chronic senescence can paradoxically promote cancer progression through sustained inflammatory signaling [48]. Treatment-induced senescence represents a double-edged therapeutic outcome: though chemotherapy and radiation can initially suppress tumor growth by inducing senescence-like arrest in cancer cells, including PGCCs, these cells may subsequently escape from senescence-like states and resume proliferation. TP53 mutations, frequently observed in PGCCs, can facilitate this senescence escape by disrupting normal growth arrest mechanisms, contributing to treatment resistance and disease relapse. This senescence-escape mechanism overlaps substantially with the dormancy–reactivation cycles observed clinically in PGCC-containing tumors, suggesting it as a potential therapeutic target to prevent cancer recurrence [49].

## 4. Treatment-Resistance of PGCCs

PGCCs demonstrate resistance to various treatments, including anti-cancer drugs and radiotherapy. Several cytotoxic agents and radiotherapy exert anti-cancer effects by blocking mitosis. Thus, these treatments cannot effectively target dormant PGCCs. In addition, multiple mechanisms underlying PGCC treatment resistance have been suggested.

Several studies have indicated that cytokine-PGCC crosstalk critically drives cancer progression and promotes chemotherapy resistance [50,51]. Cancer tissues typically feature fibrosis-rich stroma, creating a tumor microenvironment (TME) that impedes chemotherapeutic agent penetration, shields tumors from immune responses, and enhances tumor growth through growth factor expression. Migration inhibitory factors, cytokines primarily secreted by immune cells, modulate immune responses by inhibiting macrophage migration. However, PGCC-derived migration inhibitory factors facilitate immune evasion by suppressing T-cell function and promoting the expression of immune checkpoint molecules such as PD-L1 within the TME [52]. Further, PGCC-derived IL-6 stimulates fibroblasts to increase collagen production, enrich cancer-associated fibroblast (CAF) subpopulations, and enhance vascular endothelial growth factor expression. These reprogrammed CAFs promote angiogenesis and metastasis and modify the TME to favor PGCC survival [53,54].

Polyploidy enables short-term metabolic adaptations to cope with oxidative stress. Once cancer cells acquire this capability, they can survive and respond to various stressors in the TME [12,55]. The dormancy of PGCCs allows for efficient energy conservation and redistribution under poor environmental conditions [56], thereby contributing to long-term survival. It has been reported that PGCCs are rich in mitochondria, which play a crucial role in sustaining their survival by generating adenosine triphosphate through oxidative phosphorylation [57]. In addition, increased lipid droplet formation has been observed in PGCCs, suggesting that lipids are vital energy reservoirs that help these cells endure metabolic and environmental stresses [58]. The accumulation of lipids may also support membrane remodeling that facilitates PGCCs’ adaptation and persistence in the TME.

Intriguingly, Bukkuri et al. suggested that post-therapy PGCC-derived recurrent populations develop cross-resistance to multiple therapies with distinct mechanisms using mathematical modeling. The authors proposed that this may be due to PGCC memory. Though their result was theoretical and needs to be validated, it is interesting to consider treatment resistance in PGCCs [59].

For tumor-promoting effects in cell senescence, the “one-two punch” concept has been proposed as a sequential therapeutic strategy: first inducing senescence in tumor cells through conventional chemotherapy or radiation (first punch), followed by targeted elimination of these senescent cells using senolytic agents (second punch) to prevent senescence escape and tumor recurrence [60]. However, this senescence-targeting approach remains entirely hypothetical, with no preclinical validation and no clinical testing in any cancer type. Moreover, because PGCCs exhibit unique senescence-escape mechanisms and biological characteristics distinct from those of typical senescent cells, the applicability of general senescence-targeting strategies to PGCC-containing tumors is uncertain. Further research is needed to determine whether approaches developed for senescent cells could be adapted to address the clinically observed PGCC-mediated treatment resistance.

## 5. Detection Methods for PGCCs

Currently, there is no established gold standard for the diagnosis of PGCCs. The diagnostic process often requires both pathological and genetic approaches, and these facts make identifying PGCCs difficult and hinder their understanding and application in clinical practice.

### 5.1. Routine Pathology

In pathological approaches, Zhang defined PGCC nuclei as ≥ three times larger than regular-sized diploid tumor cell nuclei [2]. Although a simple and applicable definition, PGCCs represent a relatively small fraction (5–20%) of tumor cells in various cancers [61]. Furthermore, a careful cytomorphological analysis is required to distinguish true PGCCs from other giant cells, since chronic infections and inflammations also induce giant cells and can circulate in patients without cancer [62] (Figure 1) (see Section 2.2. Terminology confusion in the literature for further details).

### 5.2. Confirmatory Ancillary Methods

In the difficult situation of PGCC diagnosis, immunohistochemistry and special staining may aid in differential diagnosis. Immunohistochemical cancer stem cell markers (e.g., SOX2, OCT4, and CD44) [63] and Feulgen staining, used for semi-quantitative evaluation of DNA, can aid in the diagnosis of PGCCs.

For genetic approaches, various methods, such as flow cytometry and fluorescence in situ hybridization (FISH), have also been used. Flow cytometry and FISH offer methodological simplicity and rapid results through direct analysis of cellular DNA content without requiring cell division. Thus, they are popular methods in PGCC research.

### 5.3. Research Tools

As research tools, karyotyping and single-cell next-generation sequencing (NGS) are also used to identify PGCCs. Karyotyping is a classic method to evaluate quantitative or structural chromosomal abnormalities. Still, it is technically complex and time-consuming due to the reduced mitotic activity of PGCCs, requiring specialized expertise. Single-cell NGS is a new and beneficial technique because it provides not only PGCC ploidy and chromosomal copy numbers, but also detailed information on genomic alterations. However, it has the disadvantage of high cost.

Currently, new approaches for the diagnosis of PGCCs are being investigated. Chinen et al. examined circulating PGCCs in blood samples from patients with six types of cancer (colon, gastric, lung, breast, anal canal, and kidney). PGCCs were identified in 46 (20.18%) of 228 patients, including 14.47% of 152 non-metastatic cases and 29.85% of 67 metastatic cases [64]. Several studies have used machine learning for risk stratification of various solid tumor types based on morphological findings of cancer cells [65,66,67,68]. These studies indicated that the presence of PGCCs was a poor prognostic factor; however, detailed information about polyploid cancer cells was often not provided.

## 6. Clinical Implication of PGCCs

As described above, basic research has shown that PGCCs can drive cancer progression and promote chemotherapy resistance. PGCCs are potentially associated with more aggressive behavior and a worse prognosis. In fact, several clinical studies have reported the clinical characteristics of PGCC-containing tumors and suggested their association with more aggressive behavior in various types of solid tumors (Table 1).

Before discussing clinical implications, it is important to acknowledge limitations of the current evidence base. The prognostic value of PGCCs is based predominantly on retrospective, observational studies with heterogeneous definitions and relatively small sample sizes. Critically, comparative studies evaluating PGCCs alongside established prognostic markers such as tumor grade, stage, Ki-67 index, and TP53 status are lacking. While several studies have demonstrated associations between PGCCs and a poor prognosis, whether PGCCs provide independent prognostic value beyond these well-validated markers remains unclear, representing a significant knowledge gap that limits clinical implementation. Additionally, the lack of standardized, clinically available diagnostic criteria for PGCC identification has made prospective validation studies challenging to conduct, contributing to the predominantly retrospective nature of available evidence. Despite these methodological limitations, the consistency of findings across multiple cancer types and independent research groups suggests meaningful clinical associations that warrant further investigation.

### 6.1. Prostate Cancer

If a predominant proportion of the tumor consists of PGCCs, pleomorphic carcinoma of the prostate is diagnosed. This rare and highly aggressive variant of prostate cancer is characterized by marked pleomorphism. Comprehensive data on the frequency of this cancer are limited, and as a result, the molecular mechanisms and optimal treatment strategies remain poorly understood. However, several studies have reported a poor prognosis for this cancer. Alharbi et al. reported 30 patients with pleomorphic carcinoma of the prostate. The PGCC components were focal (<5%), but they indicated aggressive disease, rapid progression, and a poor prognosis. All patients had the usual acinar prostatic adenocarcinoma with a Gleason score of 9–10. In patients with no prior prostate cancer diagnosis and >1-year follow-up, 37% died within a median of 8 months. In those with recurrent disease, 57% died within a median of 7 months [76]. Trabzonlu et al. also reported that PGCCs are a significant prognostic factor for metastasis-free survival in a case–cohort of intermediate- or high-risk men who underwent radical prostatectomy. The number of PGCCs was a significant prognostic factor for metastasis-free survival after adjusting for the Cancer of the Prostate Risk Assessment Postsurgical (CAPRA-S) score, which incorporates the pathologic Gleason score, pathological stage, surgical margin status, and preoperative prostate-specific antigen (PSA) (adjusted hazard ratio [HR] per 1 PGCC increase, 2.00; 95% confidence interval [CI], 1.40–2.87) [69]. Schmidt et al. analyzed bone marrow aspirates from 44 patients with advanced prostate cancer and found that the presence of circulating large nuclei tumor cells (CTCs) with increased genomic content was significantly associated with poorer progression-free survival (log-rank *p* = 0.0095) [77]. In addition, single-cell copy number profiling of CTCs with increased genomic content showed clonal origins shared with typical CTCs, suggesting complete polyploidization. These findings are consistent with the features of PGCCs.

### 6.2. Lung Cancer

In the lung, pleomorphic carcinoma is defined by the WHO classification as a tumor with ≥10% PGCC (pleomorphic or spindle giant cell) components. These rare tumors (0.4–1.6% of lung malignancies) primarily affect heavy-smoking men, respond poorly to chemotherapy, and have worse outcomes than other NSCLCs [78]. One epidemiological study analyzed 461 patients with pleomorphic carcinoma from 2004 to 2014. The median OS was 9 months, and multivariate Cox analysis showed that elderly patients (>66 years) and advanced disease were predictors of poor prognosis in terms of OS. Surgery significantly improved OS in patients with localized and regional stages, but it had little impact on those with distant-stage disease. Conversely, chemotherapy reduced the risk of death in distant-stage patients, but it did not benefit those with localized or regional-stage disease. Radiation therapy had no significant effect on OS [70]. Regarding chemotherapy, small, retrospective studies have reported that pleomorphic carcinoma of the lung responded poorly to chemotherapy, mainly cytotoxic agents, and patient prognosis remained poor [79,80]. EFGR is one of the most important treatment targets in NSCLC. Several studies reported that the frequency of *EGFR* mutation is approximately ≤20%, almost equivalent to conventional NSCLC. However, the effect of EGFR inhibitors on pleomorphic carcinoma with *EGFR* mutation has been limited [81,82,83].

Genomic and immunohistochemical analyses of pleomorphic carcinoma of the lung showed key molecular characteristics. A sequencing study of 78 specimens from 52 patients identified *TP53* (71%) as the most frequently mutated gene, followed by *KRAS* (27%) and *EGFR* (8%) [83]. Both epithelial and sarcomatoid components shared activating driver mutations, with no significant differences in tumor mutational burden. Another study analyzing PD-L1, EMT-related proteins (E-cadherin, vimentin, ZEB-1), and c-MET in 16 surgically resected pleomorphic carcinoma patients found PD-L1 expression in 88% of carcinomatous areas, with 56% showing high expression [84]. PD-L1 expression in sarcomatous areas was comparable to that in carcinomatous areas; notably, high expressions of PD-L1, ZEB-1, and c-MET in sarcomatous areas correlated with a poor prognosis.

### 6.3. Pancreatic Cancer

If a predominant proportion of the tumor consists of PGCCs, undifferentiated carcinoma of the pancreas is diagnosed. It accounts for 0.3–7% of malignant pancreatic tumors. According to the WHO classification [85], undifferentiated carcinomas are classified into three subtypes: anaplastic undifferentiated carcinoma, sarcomatoid undifferentiated carcinoma, and carcinosarcoma. PGCCs correspond to anaplastic undifferentiated carcinoma, and the other two subtypes are extremely rare and not discussed here.

In one report using registry data, the median age at diagnosis was 62.0 years, and undifferentiated carcinoma showed a male predominance (71.4%). Thus, undifferentiated carcinoma shares characteristics with its counterpart, pancreatic cancer. In contrast, patients with undifferentiated carcinoma showed significantly shorter OS than those with pancreatic cancer (unadjusted HR, 1.9; 95% CI, 1.7–2.1) [71]. Undifferentiated carcinoma is recognized as a chemoresistant disease. A retrospective cohort study showed limited efficacy, with an objective response rate of 10% and median PFS of 1.84 months for first-line treatment [72]. Genomic alterations in *KRAS*, *TP53*, *CDKN2A/B*, and *SMAD4* are commonly observed in undifferentiated carcinoma. Of them, *KRAS* mutations are the most frequent, occurring in 60–80% of patients, supporting the concept that undifferentiated carcinoma originates from pancreatic cancer. In addition, EMT-related changes, characterized by E-cadherin downregulation and upregulated expression of Slug, Twist1, and ZEB-1, are frequently observed on immunohistochemistry [15].

### 6.4. Hepatocellular Carcinoma

Typically, polyploidy is associated with cancer and increases in various liver diseases that promote tumorigenesis. However, various studies have shown that the relationship between hepatocyte polyploidy and tumorigenesis in hepatocellular carcinoma (HCC) is more complicated. Lin et al. showed that polyploid hepatocytes maintain the ability to regenerate liver tissues during chronic damage without generating mitotic errors, and higher polyploidy can protect them, developing fewer HCCs following chronic liver injury [86]. Meanwhile, Matsumoto et al. demonstrated that polyploid hepatocytes are not fully protected from oncogenesis, and ploidy reduction can promote cancer initiation through multipolar mitosis, inducing chromosomal instability [87]. Binuclear polyploid hepatocytes comprise the majority of polyploid cells in normal human liver tissue, but Bou-Nader et al. showed that binuclear polyploid hepatocytes were significantly decreased during HCC progression. In comparison, mononuclear polyploid hepatocytes increased. They suggested that mononuclear polyploid hepatocytes are associated with low differentiation, high proliferation, and a poor prognosis in HCC, particularly amplified in poorly differentiated tumors with *TP53* mutations [88]. In cancer cells, Matsuura et al. found that polyploidy was detected in 36% of HCCs and identified an aggressive subset of HCC [73]. The polyploid HCCs frequently exhibited large nuclei and were associated with elevated serum alpha-fetoprotein levels, poor differentiation, and a worse prognosis than near-diploid HCCs. These characteristics suggest that polyploid HCCs share features with PGCCs.

### 6.5. Colorectal Cancer

The pathological presence of PGCCs is associated with poor differentiation, invasion, metastasis, and, consequently, poor prognosis in colorectal carcinoma (CRC). Zhang et al. investigated the clinicopathological characteristics of PGCCs in CRC [89]. More PGCCs were observed in poorly differentiated CRC than in well-differentiated CRC (well-differentiated, 27.5%; moderately differentiated, 50%; poorly differentiated, 90.2%), and PGCCs have a high predictive value for lymph node metastasis in poorly differentiated CRC. In CRC, pathological micropapillary carcinoma patterns and tumor budding (isolated small groups of cancer cells from the invasive tumor margin) are known to be factors associated with poor prognosis. PGCCs were observed in most micropapillary carcinoma patterns and tumor budding [90]. Chinen et al. indicated that the presence of PGCCs had a negative impact on patient prognosis. They showed that CRC patients with PGCCs had a significantly shorter OS than those without (*p* = 0.033) using circulating PGCCs in blood samples [64].

As in vitro data, clinical studies of locally advanced rectal cancer have indicated that neoadjuvant chemoradiotherapy (nCRT) can induce the formation of PGCCs, whose daughter cells exhibit strong migratory, invasive, and proliferative abilities. Fei et al. analyzed 304 samples from patients treated with nCRT and 301 paired samples from those without nCRT [22]. The number of PGCCs was significantly higher in tumor tissues from patients who underwent nCRT than in those who did not. In addition, more PGCCs were observed in anastomotic recurrent rectal cancer after nCRT than before treatment.

### 6.6. Urothelial Carcinoma

In urothelial carcinoma, PGCCs (pleomorphic giant cells) are an aggressive variant and are associated with poor prognosis. Portugal-Gaspar et al. reported the clinicopathological characteristics [74], indicating that all cases were associated with high-grade urothelial carcinoma. The presence of PGCCs was an independent factor associated with poor OS (HR adjusted for T stage, 2.222; 95% CI, 1.126–4.384).

### 6.7. Other Types of Cancer

#### 6.7.1. Cytomegalovirus-Related Malignancies (Breast and Ovarian Cancers)

Emerging evidence suggests that cytomegalovirus (CMV) may play a role in oncogenesis, particularly in breast cancer and high-grade serous ovarian cancer. CMV infection induces PGCC formation in these CMV-associated cancers. Recent studies have demonstrated strong correlations among CMV, PGCCs, and EZH2 expression in CMV-associated cancers, highlighting that oncogenic CMV strains induce polyploidy through EZH2 and Myc upregulation [91,92].

#### 6.7.2. Other Solid Tumors

PGCCs have been identified as a factor associated with poor OS in various cancers. In angiosarcoma, PGCCs exhibit genomic similarities to regular-sized cells. They were detected in 41.4% of patient samples and were associated with poor chemotherapy response rates (25.0% vs. 73.3%, *p* = 0.0213). In addition, PGCCs independently contributed to worse OS after adjustment for clinicopathological factors including tumor grade (adjusted HR, 2.20; 95% CI, 1.17–4.15) [75]. Similarly, one study of laryngeal cancer demonstrated that high PGCC expression was associated with worse OS [93].

Although no survival data were presented, a higher number of PGCCs was detected in more advanced stages of various cancers. In malignant melanoma, PGCC numbers were significantly higher in larger tumors than in smaller ones and in tumors with lymph node metastasis than in those without metastasis [94]. This trend was also observed in glioma, in which higher malignant grades exhibited increased PGCCs [95,96]. Similarly, in breast cancer, PGCC numbers correlated with malignancy grade: metastatic disease showed the highest PGCC numbers compared with primary breast cancer with lymph node metastasis, without lymph node metastasis, and benign disease [97].

## 7. Treatment

Current therapeutic approaches related to PGCCs can be categorized into three distinct evidence levels, each with important limitations that readers should understand when interpreting therapeutic potential.

### 7.1. Preclinical Evidence

The preclinical approaches described below represent early-stage research specifically targeting PGCC characteristics. It is important to note that these strategies remain in experimental phases, and their clinical efficacy has not yet been established. Translational research has reported several agents, including tocilizumab, mTOR inhibitors, zoledronic acid, and aurora kinase inhibitors [54,57,98,99]. However, the efficacy of these agents against PGCCs in clinical settings remains questionable based on clinical data. For example, the aurora kinase inhibitor alisertib, which potentially mediates cellular division of PGCCs, failed to demonstrate an antitumor effect against peripheral T-cell lymphoma in a phase 3 trial [100]. Similarly, tocilizumab and zoledronic acid have not been shown to have antitumor effects.

### 7.2. Retrospective Clinical Observations

The following clinical observations represent retrospective analyses of treatment outcomes rather than therapies specifically designed to target PGCCs. These findings suggest potential associations between certain treatments and PGCC behavior, but they should be interpreted cautiously as correlational rather than causal evidence. Furthermore, treatment data for patients with PGCCs are limited. However, retrospective studies indicated that taxane-based chemotherapy is a reasonable option for the treatment of patients with PGCC-containing tumors. In PGCC-containing tumors of the lung (pleomorphic carcinoma), one small retrospective study reported that the paclitaxel-containing regimen, carboplatin plus paclitaxel, or carboplatin plus nab-paclitaxel, offered a high objective response rate of 60.0% and a median OS of 16.7 months [101]. Similarly, in PGCC-containing tumors of the pancreas (undifferentiated carcinoma), a paclitaxel-containing regimen was associated with a significant OS benefit in patients with advanced disease (HR, 0.221; 95% CI, 0.076–0.647) [72]. Although retrospective in nature, these results indicated that a paclitaxel-containing regimen for PGCC-containing tumors may provide promising results. Thus, we are currently conducting a phase 2 clinical trial of gemcitabine plus nab-paclitaxel for PGCC-containing tumors (jRCTs031220099).

### 7.3. Theoretical Considerations

The following approaches represent theoretical concepts derived from PGCC biology, but they remain entirely hypothetical. These strategies require extensive preclinical validation before any clinical consideration. Today, immune checkpoint inhibitors (ICIs) have emerged as a new treatment paradigm for patients with many types of cancer [102]. High microsatellite instability is considered a biomarker for predicting the efficacy of immune checkpoint inhibitors. Notably, tumors tend to exhibit either chromosomal instability or high microsatellite instability [103], indicating that tumors with chromosomal instability typically display low microsatellite instability. The potential response of PGCCs to ICIs remains unclear, with no clinical data specifically evaluating ICI efficacy in PGCC-containing tumors. While theoretical concerns exist regarding immunosuppressive microenvironments associated with PGCCs, clinical validation is needed to determine whether these mechanistic considerations translate to altered therapeutic response.

As previously described, p53 is a tumor suppressor protein and a key regulator of PGCCs. Thus, p53 is a potential therapeutic target. Recently, a novel p53 reactivator, rezatapopt, showed a promising antitumor effect on *TP53 Y220C*-mutated tumors [104]. This small molecule binds to the *TP53 Y220C* mutant p53 protein, restoring its tumor-suppressor activity. Although this drug’s development is still in the early stages, it has the potential to be effective against PGCCs.

Another potential therapeutic target is EZH2, which plays a critical role in the polyploidization of CMV-associated cancer cells. Recent studies have shown that EZH2 inhibition reduces malignant transformation in CMV-associated breast cancer and high-grade serous ovarian cancer [91,92], suggesting that EZH2 is a promising therapeutic target for CMV-associated malignancies. The EZH2 inhibitor, tazemetostat, showed encouraging results in follicular lymphoma [105] and advanced epithelioid sarcoma with loss of INI1/SMARCB1 in phase 2 trials [106].

## 8. Conclusions

PGCCs represent a clinically significant but underexplored aspect of tumor biology. PGCC-containing tumors exhibit marked treatment resistance and are associated with a poor prognosis across multiple solid tumor types, including prostate, lung, and pancreatic cancers. Despite growing recognition of their clinical importance, translating PGCC research into clinical practice requires a systematic approach across three phases.

First, the foundation for clinical implementation requires establishing standardized pathology criteria for PGCC identification and reporting. This includes defining minimum thresholds for PGCC percentage, standardized measurement protocols, and consistent morphological criteria across institutions. Complementary molecular markers should be developed to distinguish true PGCCs from morphological mimics, incorporating techniques such as DNA content analysis, chromosome enumeration, and gene expression profiling to ensure diagnostic accuracy.

Second, rigorous clinical validation through multicenter studies is essential to establish the prognostic value of PGCCs. These studies must include a direct comparison with established markers, such as tumor grade, stage, Ki-67 index, and TP53 status, to determine their independent prognostic value. Based on these findings, PGCC-based risk stratification models should be developed and validated to guide clinical decision-making.

Finally, clinical trials testing PGCC-targeted therapeutic strategies represent the ultimate translational goal. These should encompass biomarker-driven patient selection for specific treatments, evaluation of combination therapy approaches for PGCC-containing tumors, and assessment of novel therapeutic targets such as p53 reactivation and epigenetic modulators. By systematically implementing this roadmap, PGCCs may transition from intriguing biological phenomena into actionable clinical biomarkers that improve patient outcomes.

## Figures and Tables

**Table 1:** Summary of prognosis of polyploid giant cancer cell-containing solid tumors.

Author (Year)	Population	Study Type	No. of Patients	Survival Metric	Median Survival Time	Comparison	Survival Comparison Summary
Prostate cancer (Key genetic alterations: *ETS*, *PTEN*, *TP53*, *AR*)							
Trabzonlu (2023) [69]	Prostate cancer with PGCCs with intermediate- or high-risk	Retrospective observational study	239	Metastasis-free survival	–	Prostate cancer with PGCCs vs. control prostate cancer	Adjusted HR, 2.00 (95% CI, 1.40–2.87) *
Lung cancer ^†^ (Key genomic alterations: *EGFR*, *KRAS*, *ALK*, *MET*)							
Chen (2022) [70]	Registry data	Population-based study	461	OS	9 months		
Pancreas (Key genomic alterations: *KRAS*, *TP53*, *SMAD4*, *CDKN2A*)							
Clark (2012) [71]	Registry data	Population-based study	353	OS	3 months	Undifferentiated carcinoma vs. control pancreatic cancer	Unadjusted HR, 1.9 (95% CI, 1.7–2.1)
Imaoka (2020) [72]	Treated with chemotherapy	Retrospective observational study	50	OS	4.08 months		
HCC (Key genomic alterations: *TERT*, *TP53*, *CTNNB1*, *ARID1A*)							
Matsuura (2023) [73]	HCC with PGCCs	Retrospective observational study	20	OS	–	HCC with PGCCs vs. near-diploid HCC	log-rank *p* = 0.013
Colorectal cancer (Key genomic alterations: *APC*, *TP53*, *KRAS*, *PIK3CA*)							
Chinen (2024) [64]	Presence of circulating PGCCs	Retrospective observational study	9	OS	–	Presence of circulating PGCCs vs. absence of circulating PGCCs	log-rank *p* = 0.033 (5-years OS rate 76% vs. 96%)
Urothelial carcinoma (Key genomic alterations: *TP53*, *TERT*, *PIK3CA*, *RB1*)							
Portugal-Gaspar (2024) [74]	High-grade urothelial carcinoma	Retrospective observational study	21	OS	–	Urothelial carcinoma with PGCCs vs. conventional urothelial carcinoma	Adjusted HR, 2.22 (95% CI, 1.126–4.384)
Angiosarcoma (Key genomic alterations: *TP53*, *MYC*, *POT1*, *KDR*)							
Tan (2021) [75]	Treated with chemotherapy	Retrospective observational study	22	OS	9.6 months	Presence of PGCCs vs. absence of PGCCs	Adjusted HR, 2.20 (95% CI, 1.17–4.15)

For consistency, all survival time have been converted to months in this review. HR > 1 indicates that PGCC-containing tumors have a poorer prognosis. * HR per 1 PGCC increase. Lung cancer ^†^, non-small cell lung cancer. HR, hazard ratio; CI, confidence interval; OS, overall survival; HCC, hepatocellular carcinoma; PGCCs, polyploid giant cancer cells.

## Data Availability

No new data were created or analyzed in this study. Data sharing is not applicable to this article.
